# High School Educator Training by Simulation to Address Emotional and Behavioral Concerns in School Settings: A Randomized Study

**DOI:** 10.1007/s41347-022-00243-9

**Published:** 2022-03-26

**Authors:** Glenn Albright, Mina Fazel, Nikita Khalid, Jeremiah McMillan, Don Hilty, Kristen Shockley, Shashank Joshi

**Affiliations:** 1 Department of Psychology, Baruch College, City University of New York, One Bernard Baruch Way, New York, NY 10010 USA; 2 Medical Services Division, Department of Psychiatry, University of Oxford, Oxford, UK; 3 Educational Psychology, Graduate Center, City University of New York, 365 5th Avenue, New York, NY 10016 USA; 4 Department of Psychology, University of Georgia, 125 Baldwin Street, Athens, GA 30602 USA; 5 Department of Psychiatry and Behavioral Sciences, University of California, 2230 Stockton Boulevard, DavisSacramento, CA USA; 6 Division of Child & Adolescent Psychiatry, Stanford University, 401 Quarry Road, Stanford, CA 94305-5719 USA

**Keywords:** Adolescent health, Mental health, Prevention, Suicide, Simulations

## Abstract

The purpose of this study is to examine the impact of an online virtual human role-play simulation in teaching high school educators and staff to identify, talk to, and if necessary, refer students in psychological distress to support services. High school educators (*N* = 31,144) from 43 US states and 5 American territories completed a baseline survey and then randomly assigned to a wait-list control or treatment group. Participants in the treatment group completed the training simulation which included active learning strategies to teach evidenced-based communication strategies such as motivational interviewing to build skills and shift attitudes. Immediately after the training, treatment group participants completed a post-survey and then a 3-month follow-up survey. Baseline and post-surveys included the validated gatekeeper behavior scale measures which assess attitudinal constructs that predict helping behaviors. Self-reported helping behaviors were collected at baseline from both groups and at the 3-month follow-up for the treatment group. The treatment group participants’ post and follow-up data were compared to the control group’s baseline measures. The treatment group post-training scores were significantly higher (*p* < .001) than the control group’s baseline scores for all gatekeeper behavior scale attitudinal constructs of preparedness, likelihood, and self-efficacy to engage in helping behaviors. A teacher subsample reported significant increases (*p* < .001) in the number of students referred to mental health support services when compared to baseline measures of the control group. Role-play simulations hold promise in teaching educators to become the “eyes and ears” of student mental health by empowering them to identify students in psychological distress, engage them in effective conversations about their concerns, and if necessary, make a referral to behavioral health support services. Future studies need to implement measures that document students entering counseling as a result of self-reported referrals and examine the impact of the training on the overall mental health culture within schools. Such studies could lead to simulations being widely adopted to support public health initiatives that address student mental health and wellness.

## Introduction

The need for effective gatekeeper training addressing student mental health problems and related behavioral challenges has never been more pronounced. According to the US Youth Risk Behavior Surveillance Survey, 30% of students nationwide reported that during the prior 12 months, they had felt so sad or hopeless (almost every day for 2 or more weeks in a row) that they stopped doing some usual activities (Kann et al., 2016). The suicide rate among US adolescents has tripled over the past 60 years, becoming the second leading cause of death in this age group (Eaton et al., 2012; Kann et al., 2016) with similar increases observed in other high-income countries (Cha et al., 2018). This is further complicated by the impact of the COVID-19 pandemic and its relationship with increasing mental health problems and psychosocial difficulties (Fegert, et al., 2020; World Health Organization, 2020). Unfortunately, many barriers exist in accessing mental healthcare services (Fazel et al., 2014; Loewen, 1993) which include cultural factors that reinforce stigma or perceived stigma in particular groups, such as those that emphasize individualism and self-reliance, low interpersonal dependency, and discomfort with emotions and their relationship with gender norms (Komiya et al., 2000). When combined, barriers contribute to only one-third (36%) of US adolescents with mental disorders receiving services for their illness (Merikangas et al., 2010). Furthermore, based on the US National Comorbidity Survey (Merikangas et al., 2010), fewer than one in five affected adolescents received services for anxiety, eating, or substance use disorders, with marked racial disparities; findings that are similar to those in other high-income nations such as in Europe, Canada, and Australia (Fazel et al., 2014; Malla et al., 2016). Yet, the evidence in supporting early mental healthcare treatment has clearly established that adolescents who accessed treatment have better educational and mental health outcomes in young adulthood starting at 18 years of age (Neufeld et al., 2017). Additionally, early treatment can impact teachers with high levels of burnout (i.e., lower levels of career commitment and job satisfaction) due to the link between teacher stress and students’ problematic behaviors (e.g., vandalism, aggression, verbal abuse, and other challenges) and dealing with parents of disruptive students (Fore et al., 2002; Maslach et al., 1986; Otero-Lopez et al., 2009). Thus, it stands to reason that when effective gatekeeper skills are applied and students are referred to support services, teacher burnout can potentially be reduced.

While school interventions by counselors, nurses, and mental health professional are often used as a marker of improved access, resources may be limited, especially during the pandemic; thus, task-shifting or building skills, attitudes, and knowledge in the teaching workforce may have broader impact on students-similar to training primary care providers or pediatricians to provide mental health services across the broader population. Three areas of intervention appear promising. First, gatekeeper training involves teaching individuals to recognize the signs of psychological distress, to approach and talk with those they are concerned about, and if necessary and available, to make a referral to mental health support services. In general, gatekeeper training programs have demonstrated increases in knowledge and self-efficacy, but relatively few studies have examined changes in gatekeeper behaviors (Arensman et al., 2016; Gask et al., 2017; Hangartner et al., 2019; Lamis et al., 2017; Mo et al., 2018; Osteen, 2018; Rallis, 2017; Rallis et al., 2018; Reiff et al., 2019; Sylvara & Mandracchia, 2019; Terpstra et al., 2018; Yeates, 2019). Another area of exploration that has gained traction is using virtual human role-plays for developing evidence-based communication skills such as motivational interviewing (MI)-“virtual humans (VH) are defined as automated, three-dimensional agents that converse, understand, reason, and exhibit emotions-to leverage conversations to drive behavioral and attitudinal change” (Albright et al., 2016a). MI is a goal-oriented, person-centered counseling approach designed to help people resolve their ambivalence about behavior change in a supportive, collaborative style (Miller & Rollnick, 2013). MI involves strategically evoking participants’ thoughts and feelings, to explore internal conflicts and to build upon or amplify existing motivational resources. This is done by (1) asking open-ended questions, (2) providing affirmation, (3) reflective listening (listening closely and selectively emphasizing the person’s statements), and (4) summarizing the person’s self-assessments (Miller & Rollnick, 2012). Teaching MI skills with gatekeeper virtual role-play simulations has been successfully demonstrated in several studies (Albright et al., 2018; Bradley & Kendall, 2019; Bradley et al., 2019; Coleman et al., 2019; Long et al., 2018; Pasco et al., 2012; Rein et al., 2018; Vallance et al., 2014).

Lastly, the advantages of using VHs in role-plays are numerous. This includes learners feeling less judged, social evaluative threat, and embarrassment when compared to face-to-face role-plays with instructors and/or peers, which can lead to reduced learning, retention, and negative emotions (Brom et al., 2016; Cooper et al., 2018; Jouriles et al., 2011; Liew et al., 2014; Mesagno et al., 2012; Plancher et al., 2019; Plass & Kalyuga, 2019; Smallwood et al., 2009; Taylor, 2018; Van Ast et al., 2014). Also, VH simulations can support high fidelity which results in accurate knowledge dissemination, the elimination of trainer bias, and dashboards that can deliver reliable performance feedback (Albright et al., 2016a). Plus, the complex algorithms within game engines that drive role-play simulations can provide each learner with a unique and realistic experience because VHs can continually respond in the most efficacious way to promote skill development. Lastly, the fact that learners find it easier to talk to and open up with VHs and are less concerned about making mistakes (along with other advantages) cannot be understated (Fiske et al., 2019; Hart & Proctor, 2019; Kang & Gratch, 2010; Lucas et al., 2014; McGaghie et al., 2009; Rizzo et al., 2016; Robb et al., 2015).

## Objectives

The objectives of this study are to examine the effectiveness of an online virtual role-play simulation designed to teach high school educators and staff to (1) identify students in psychological distress, (2) approach students they are concerned about and engage them in a conversation using evidenced-based MI communication strategies, and (3) make a referral to mental health support services if necessary. Specifically, we hypothesize that the training will result in (1) high satisfaction ratings; (2) a significant increase in the belief that part of the role of educators is to connect students experiencing psychological distress with mental health support services; (3) significant pre- to post-intervention improvements in high school educators’ attitudes of preparedness, likelihood, and self-efficacy to identify signs of student psychological distress, talk to students about concerns, and make a referral to mental health support services; and (4) a significant change in self-reported gatekeeper behaviors 3 months following completion of the training that include identifying students in psychological distress, approaching and talking with them about concerns, and making referrals to mental health support services.

## Methods

### Simulation Overview

*At-Risk for High School Educators*, was developed by Kognito (www.kognito.com) and is listed in Section III of the US Suicide Prevention Resource Center’s Best Practices Registry for Suicide Prevention (2012) and included in the US Substance Abuse and Mental Health Services Administration’s (SAMHSA) National Registry of Evidence-Based Programs and Practices (2016). In the simulation, participants enter an online environment where they practice role-playing with emotionally responsive virtual students coded with memory, personality, and will respond like real students in psychological distress. For each conversation, participants need to create a safe environment, gain the virtual student’s trust, and gather enough information to determine what the perceived psychological distress is by using specific MI strategies that are imbedded into the active learning experience.

Participants communicate with the virtual students by selecting from a dynamic menu of dialogue options. The dialogue options represent a variety of effective, neutral, and ineffective conversation tactics determined by nationally recognized subject matter experts and end-users. In some cases, a tactic that is ineffective at one point in the conversation may be effective elsewhere. Once participants choose a dialogue option, they see their virtual character ‘‘perform’’ the dialogue and then observe the verbal and nonverbal response of the virtual student. A new set of dialogue options then appears, based on which tactic was selected. A virtual coach provides real-time positive feedback for correct tactics and makes suggestions for tactics less likely to improve communication such as being judgmental, critical, or making a diagnosis. Each conversation continues to build on the MI strategies they used previously to scaffold the learning.

The first role-play is with a student who is experiencing anxiety, is suspected of cutting, became overwhelmed when hearing she got a “B” on an exam, and has been texting the yearbook committee teacher at all hours at night asking questions. The second is with a student who is academically at-risk, has poor attendance, outbursts of anger, and is suspected of illicit drug use and bullying. The last role-play is with a student who is shy, withdrawn, and revealed possible suicidal thoughts in an essay, which turns out to be related to his father dying by suicide.

The role-plays are completed once the participants earn the student’s trust, who then reveal what is creating the psychological distress, which ultimately leads to recommendations and a referral. The participant then has access to a dashboard after each conversation which provides an overview of the student’s signs of distress, how they did in managing the conversation, advice on how to refer other students with similar symptoms, and a reminder to follow-up with the student. When participants successfully conclude all three conversations, they are provided with a printable summary of best practices and a certificate of completion. Lastly, the simulation includes a wide range of didactic resources such as customized referral information for local resources, a summary of course content and links to national resources such as the National Suicide Prevention Lifeline, Suicide Prevention Resource Center’s teachers resource page, and stop bullying information.

### Design

**Fig. 1:**
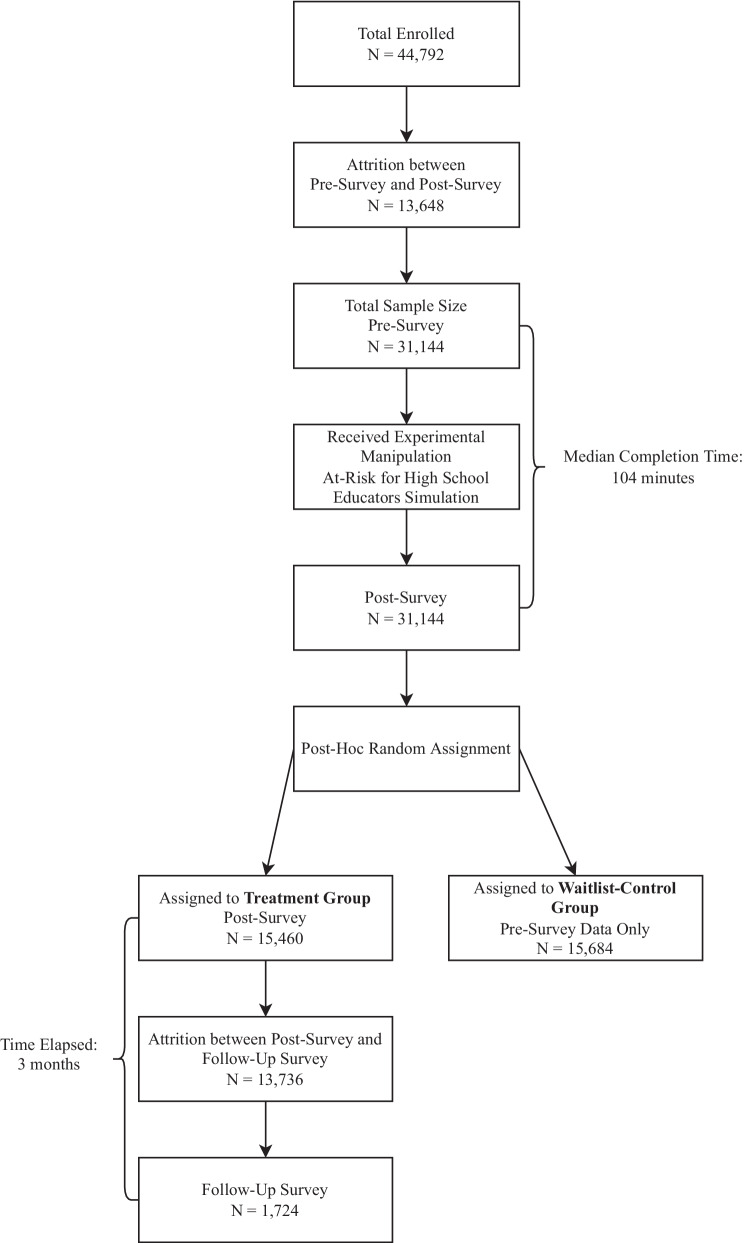
Consort diagram of study flow

All participants agreed to an informed consent, and upon completing a baseline survey, were randomly placed into either the treatment or wait-list control groups. Treatment group participants completed the simulation, and immediately afterwards a post-survey, and 3-months later, a follow-up survey. The treatment group participants’ post and follow-up data were compared to the control group’s baseline measures (see Fig. 1 for study flow consort diagram). All training and data collected by the surveys were from computers of the participant’s choice in order to maximize a private and structured learning environment and could therefore be in their office, home, or other environment. The Baruch College Human Research Protection Program/Institutional Review Board determined that no ethics approval was required for this study.

### Participants

The original sample consisted of 44,792 participants recruited by email from district superintendent offices, principals, or word-of-mouth and accessed the training at no cost as it was provided by a state or local government entity such as the state department of health or education, county health department, or school district. We excluded 13,648 teachers and staff who did not complete the study, leaving a final sample size of 31,144 from 43 US states and five American territories (79.8% in the US State of Texas) to be randomly assigned to a treatment or waitlist control group. All participants who were dropped from the study were significantly higher on baseline measurements of preparedness (*p* < 0.01; Cohen’s *d* = 0.03), likelihood (*p* < 0.05, Cohen’s *d* = 0.02), and self-efficacy (*p* < 0.001; Cohen’s *d* = 0.04). In the final sample, 15,684 were randomly assigned to a waitlist control group, and 15,460 were assigned to a treatment group. In total, the median time for participants in the treatment group to complete the baseline survey, the training simulation, and that post survey was 104 min.

### Measures

All measures were based on Kirkpatrick’s training evaluation model (Kirkpatrick, 1996; Kirkpatrick & Kirkpatrick, 2006). This model comprises four levels and includes level one satisfaction; level two learning, impact on attitudes, knowledge, and/or skills; level three behavior, represents any changes in behavior; and level 4 results, the overall long-term benefits derived from the training such as a shift in student mental health culture or return on investment. The learning stage is important to evaluate because behavior will not change if the desired attitudes, knowledge, and skills are not acquired. The fourth level was not assessed as it was not within the scope of this study.

Level one measures were included in the post-training survey and included levels of satisfaction and means efficacy. Means efficacy is a measure of an individual’s belief in the utility of the tools available for performing a job and has been correlated with changes in behavior (Eden et al., 2010). Specifically, level one items included:

1. Overall, how would you rate the course? (4-point Likert scale from “poor” to “excellent”)
2. Would you recommend the simulation to a colleague? (yes or no)
3. All educators in their institution should take the training. (yes or no)
4. To what extent do you think that the course is (means efficacy-5-point Likert scale from “not at all or to very little extent” to “a very great extent”):
5. A useful tool?
6. Well-constructed?
7. Easy to use?
8. Likely to help you with troubled students?
9. Based on scenarios that are relevant to you and your students?

Level two measures were administered at baseline and in the post-survey and included the gatekeeper behavior scale (GBS). The GBS is a validated 11-item tool used to determine the impact of online gatekeeper simulations (Albright et al., 2016b). The GBS measures attitudes and intentions that have been shown to be related to changes in gatekeeper behaviors and include three dimensions or subscales: participant’s preparedness, likelihood, and self-efficacy to engage in gatekeeper behaviors such as identifying students in psychological distress, engaging them in a conversation about their concerns, and making a referral to support services. The preparedness composite is comprised of five items, the likelihood or behavioral intent two items, and self-efficacy four items (for individual items and Likert scales see Appendix 1). In this study, participant preparedness was computed as the average of five separate items (Cronbach’s α = 0.91), likelihood as the average of two separate items (Cronbach’s α = 0.78), and self-efficacy as the average of four separate items (Cronbach’s α = 0.89).

Two additional level two measures assessed included (1) belief that part of the role of educators is to connect students experiencing psychological distress with mental health support services and( 2) participants being able to recognize signs of psychological distress in themselves. Both measures were rated on a 4-point Likert scale ranging from “strongly disagree” to “strongly agree.”

Level three measures of behavior were obtained in two ways. First, participants were asked in the 3-month follow-up survey, if as a result of the training there were increases in the number of students: (1) recognized as exhibiting signs of psychological distress, (2) approached to discuss concern, and (3) referred for mental health support. Responses were reported based on a 4-point Likert scale from “strongly disagree” to “strongly agree.” The second measurement involved participants reporting in the baseline and follow-up surveys the approximate number of students over the past 2 academic months they had (1) been concerned about due to their psychological distress, (2) approached to discuss concerns about their psychological distress, and (3) referred to school support services. This second measurement was assessed in two ways. The first was comparing the baseline behaviors of the control group to the follow-up behaviors in the treatment group, and the second was examining changes in behaviors from baseline to follow-up within the treatment group. Lastly, treatment group participants were asked at follow-up if there had been an increase in the number of conversations they had with other adults in their school community regarding students they were concerned about since completing the training. Responses were rated on a 4-point Likert scale ranging from “strongly disagree” to “strongly agree.”

All demographics were collected in the post survey and included gender, race/ethnicity, primary role, age, and years working in education.

### Analyses

In order to reduce type I error as the GBS outcomes were expected to be closely associated, a multivariate analysis (Hotelling’s *T*^2^) was utilized to assess the impact of the simulation on outcomes as a whole. Independent samples *t*-tests were used to compare control and treatment groups on each scale individually and finally to compare groups on each individual item. To determine if the treatment group experienced an increase in self-reported gatekeeper behaviors, two analyses were conducted. First, a set of independent samples *t*-tests were run to determine if the treatment group exhibited higher helping behaviors at follow-up than reported by the waitlist control group at baseline. Second, a paired-samples *t*-test was run to determine if these behaviors increased within the treatment group from baseline to follow-up. Before conducting the independent samples *t*-tests, analyses revealed that all behaviors were not significantly different between treatment group and control group at baseline. Lastly, a separate statistical analysis was conducted on a subsample that only included teachers to determine if this group experienced an increase in self-reported number of students helped. This was accomplished in the same two ways. First, a set of independent samples *t*-tests were run to determine if the teacher treatment group exhibited significantly higher helping behaviors than the waitlist control group, and second, a set of paired-samples *t*-tests were run to determine if these behaviors increased within the treatment group. Lastly, to address possible developer bias, all statistical analyses and results were conducted and drafted by independent consultants from the University of Georgia, USA.

## Results

Demographics show that 65% of participants were early to mid-career and white, female teachers (Table 1). Ninety-two percent were required to complete the simulation as part of their school district training requirements, and 12.0% had previously received mental health training.

**Table 1:** Participant demographic information

	*N*	**%**
Gender		
Female	19862	65.3
Male	10391	34.2
Transgender	40	0.1
Other	116	0.4
Race/ethnicity		
White, non-Hispanic	19655	66.3
Black, non-Hispanic	2392	8.1
Hispanic	6263	21.1
American Indian/Alaska Native	201	0.7
Asian	546	1.8
Native Hawaiian/Other Pacific Islander	64	0.2
Multiple ethnicities	505	1.7
Primary role		
Teacher	21132	71.6
School administrator	1669	5.7
Mental health professional/social worker	1598	5.4
Administrative assistant/clerical	1175	4.0
Other (e.g., student teacher, teacher’s aide, healthcare provider, support staff)	3927	13.3
Age	*M* = 42.28, SD = 12.11	
Years working in education	*M* = 11.07, SD = 9.55	

The control and treatment groups did not differ significantly on gender, race/ethnicity, age, years in education, work role, or previous degree of mental health training (all *χ*^2^-test and *t*-test *p*-values were greater than 0.05). Additionally, the control group and treatment group did not differ significantly on initial levels of preparedness, likelihood, or self-efficacy (all independent-samples *t*-test *p*-values were greater than 0.05). Note that sample size varies slightly for analyses below due to individual missing data.

Complete demographic information is presented in Table 1. Note that sample size varies slightly for analyses below due to individual missing data.

### Level One: Satisfaction and Means Efficacy

After completing the training, participants reported that they were highly satisfied with the simulation, with an average rating of 3.17 on a 4-point scale (37.2% of participants rated the simulation “excellent” and 43.0% as “very good”). In addition, 98% of participants agreed or strongly agreed that all educators in their school should take the simulation, and 95% indicated that they would recommend the simulation to a colleague. Additional means efficacy information about treatment group attitudes toward the simulation can be seen in Table 2. Overall, the results suggest that most participants found the simulation to be helpful and effective.

**Table 2:** Means efficacy items-percentage of participants who endorsed each option

	Not at all/very little	A little	Some	Great	Very great
*To what extent do you think that the course is:*					
A useful tool?	0.4%	1.5%	18.0%	52.6%	27.5%
Well-constructed?	0.6%	1.5%	13.8%	51.1%	33.1%
Easy to use?	0.8%	2.3%	13.9%	47.2%	35.7%
Likely to help you with troubled students?	0.6%	1.8%	17.9%	50.2%	29.6%
Based on scenarios that are relevant to you and your students?	0.8%	2.1%	17.4%	47.8%	31.9%
Able to aid you in getting timely help to your students?	0.8%	1.9%	18.3%	50.7%	28.3%

### Level Two: Gatekeeper Attitudes and Beliefs

Results from the GBS Hotelling’s *T*^2^ test indicated that the treatment group post-survey results differed significantly on the three outcome variables of preparedness, likelihood, and self-efficacy when compared to the control group baseline, *F*(3,31,057) = 2329.98, *p* < 0.001, *η*^2^ partial = 0.18.

Statistical analyses consisted of evaluating each of the three primary outcomes individually. Preparedness of the treatment group (*M* = 4.13, SD = 0.64) was significantly higher than preparedness of the control group (*M* = 3.48, SD = 0.73), *t*(31,122) = 83.65, *p* < 0.001. Likelihood of the treatment group (*M* = 3.52, SD = 0.54) was significantly higher than likelihood of the control group (*M* = 3.25, SD = 0.61), *t*(31,097) = 42.16, *p* < 0.001. Lastly, self-efficacy of the treatment group (*M* = 3.36, SD = 0.50) was significantly higher than self-efficacy of the control group (*M* = 3.02, SD = 0.57), *t*(31,080) = 55.63, *p* < 0.001. Independent-samples *t*-test results for each individual item from the preparedness, likelihood, and self-efficacy scales can be seen in Table 3.

**Table 3:** Individual scale item significance testing

	Mean-control (SD)	Mean-treatment (SD)	*t*-value
***Preparedness: How would you rate your preparedness to…***			
Recognize when a student’s behavior is a sign of psychological distress	3.50 (.76)	4.12 (.66)	76.64
Recognize when a student’s physical appearance is a sign of psychological distress	3.49 (.79)	4.12 (.67)	75.27
Discuss with a student your concern about the signs of psychological distress they are exhibiting	3.32 (.90)	4.10 (.70)	85.96
Motivate a student exhibiting signs of psychological distress to seek help	3.51 (.87)	4.13 (.69)	69.48
Recommend mental health support services to a student exhibiting signs of psychological distress	3.59 (.94)	4.18 (.70)	63.07
***Likelihood: How likely are you to…***			
Discuss your concerns with a student exhibiting signs of psychological distress?	3.18 (.66)	3.50 (.57)	44.77
Recommend mental health support services to a student exhibiting signs of psychological distress?	3.31 (.67)	3.55 (.57)	32.74
***Self-efficacy: I feel confident…***			
In my ability to discuss my concerns with a student exhibiting signs of psychological distress	3.02 (.62)	3.34 (.53)	47.81
In my ability to recommend mental health support services to a student exhibiting signs of psychological distress	3.06 (.65)	3.37 (.53)	44.89
That I know where to refer a student for mental health support	3.01 (.67)	3.39 (.54)	54.28
In my ability to help a suicidal student seek help	2.98 (.68)	3.34 (.55)	51.18
***Additional items***			
Part of the role of educators is to connect students experiencing psychological distress with mental health support services	3.27 (.59)	3.43 (.54)	25.52
I can recognize signs of psychological distress in myself	3.22 (.64)	3.42 (.54)	2.32*

All items significant at *p* < .001, except last item *p* < .05

Table 3 also shows that the treatment group had significantly higher beliefs that part of the role of educators is to connect students experiencing psychological distress to mental health support services, *t*(30,074) = 25.52, *p* < 0.001. Lastly, although it was not a direct aim of this intervention, it is interesting to note that a small subsample within the treatment group rated their ability to recognize signs of psychological distress in themselves significantly higher than the control group, t(229) = 2.32, *p* < 0.05.

### Level Three: Gatekeeper Behaviors

To assess the change in behaviors, we examined the responses of 1724 participants 3 months after completion of the training. When asked if as a result of the simulation they had changed key behaviors in assisting students in psychological distress, an average of 41.8% either “agreed” or “strongly agreed” that they had increased the number of students they recognized as in psychological distress, approached to talk to, and referred to mental health services (see Table 4). Lastly, 56.7% either agreed or strongly agreed that as a result of taking the simulation, there was an increased number of conversations they had with other adults within their school community regarding students they were concerned about.

When comparing gatekeeper behaviors in the treatment group at follow-up with the control group baseline behaviors, there was a significant increase in the number of students: (1) concerned about due to psychological distress and (2) approached to discuss this concern (see Table 5).

**Table 4:** Self-reported treatment group changes in behavior at follow-up

	Strongly disagree	Disagree	Agree	Strongly agree
***As a result of taking the course, there has been an increase in the number of:***				
Students that I recognized as exhibiting signs of psychological distress	4.2%	49.5%	39.8%	6.4%
Students that I approached to discuss my concern about their psychological distress	5.1%	52.8%	36.2%	5.9%
Students that I referred for mental health support services	6.3%	56.7%	31.7%	5.4%
Conversations I have had with other adults in my school community regarding students I am concerned about	5.2%	38.1%	47.0%	9.7%

**Table 5:** Changes in behavior as a result of the simulation

	Between treatment and control		
	Control mean (SD)	Treatment mean (SD)	*t* value
***In the past two academic months, approximately how many students have you…***			
Been concerned about due to their psychological distress?	1.95 (8.81)	2.40 (4.37)	2.01*
Approached to discuss your concerns about their psychological distress?	1.62 (8.41)	2.06 (3.98)	2.07*
Referred to school support services?	1.40 (6.25)	1.63 (3.13)	1.43

^*^p < .05

^†^*p* < .06

To further investigate the impact on referrals, data from a teacher subsample (*N* = 1120) was analyzed. There were no significant increases when comparing the control and treatment groups; however, the results show a significant increase in referrals to school support services for the within group analysis (see Table 6).

**Table 6:** Changes in behavior as a result of the simulation-teacher subsample

	Within-treatment group		
	Pre-test mean (SD)	Follow-up mean (SD)	*t* value
***In the past two academic months, approximately how many students have you…***			
Been concerned about due to their psychological distress?	1.81 (7.02)	2.00 (3.09)	.90
Approached to discuss your concerns about their psychological distress?	1.39 (6.57)	1.63 (2.29)	1.25
Referred to school support services?	1.00 (2.05)	1.22 (1.68)	3.59***

^***^p < .001

## Discussion

There are a several limitations to note that include the relatively short follow-up time and the use of self-reported data. Gaining access to the schools’ support services (counseling) referral records would have allowed us to objectively measure the impact of the intervention. In addition, some triangulation of the data with student input about their perceptions of teachers who completed the training and those who did not might further enhance the findings. This can include assessing the impact of the skills taught taking into account the perceptions of students from diverse cultural backgrounds as MI is a critical component of the simulation and a useful tool in cross-cultural communication due to its non-confrontational and supportive nature (Lundahl & Burke, 2009). It is also important to note that those participants who dropped out appear to have initially higher preparedness, likelihood, and self-efficacy scores than those who remained to complete the post-test. However, effect sizes are small, suggesting that the large sample size might be driving this finding. Even so, it is possible that the simulation may not have the same impact on non-completers, perhaps because they initially had higher skill sets to assist students experiencing psychological distress. In addition, although the study’s use of a wait-list control design with random assignment to the treatment and control group provides good internal validity, there was no comparison to traditional face-to-face professional development. However, the control group not receiving training is often the standard of preparation given the lack of teacher pre-service or in-service training. Lastly, the level one survey item on how you would rate the training is skewed positively.

Beyond the limitations, the results show that all four hypotheses were supported. The simulation significantly increased the GBS attitudes of preparedness, likelihood, and self-efficacy in participant ability to identify, talk to, and refer students in psychological distress. Participants also reported statistically significant increases in two gatekeeper behaviors at follow-up that included the number of students: (1) concerned about due to psychological distress and (2) approached to discuss this concern. A subsample of teachers reported significant increases for referrals to school support services. This enhanced role of the teacher as a natural helper and a trusted adult who conveys a sense of care and concern on school campuses is important to acknowledge as school communities enact universal suicide prevention strategies (Joshi et al., 2015, 2017). Ninety-five percent of participants also indicated that they would recommend the simulation to a colleague. Lastly, there was a significant increase in the belief that part of the role of faculty, staff, and administrators is to connect students experiencing psychological distress with mental health support services. This result coupled with 57.8% of participants stating that there was an increased number of conversations they had with other educators regarding student mental health is encouraging.

The results have significant implications for teachers. The increase in self-efficacy measures is quite noteworthy, as Bandura’s (1977) integrative framework of personal efficacy or perceived behavior control posits that self-efficacy is both a direct and indirect predictor of behavior. When self-efficacy is high and people feel confident in their abilities, this also leads to a sense of control in terms of ability to change their behavior in future circumstances. In this study, participants reported significant increases (*p* < 0.001) in their self-efficacy to (1) discuss my concerns with a student exhibiting signs of psychological distress, (2) know where to refer a student for mental health support, (3) help a suicidal student seek help, and (4) recommend mental health support services to a student exhibiting signs of psychological distress.

Teachers, school administrators, and parents have had to re-evaluate the structure and process of education as a result of the challenges spurred by COVID-19-but perhaps this is an opportunity to improve. Mental health disorders are increasing among school-aged youth, and we must be especially careful as we do not yet fully understand the mental health consequences of COVID-19. This underscores the importance of expanding school-based interprofessional in-person and virtual assessment, prevention, and interventions for mental health and illness for students and families (Collishaw, 2015; Fazel et al., 2014; Kann et al., 2016). Professional development is an investment-and this requires time, expense, and other costs-but it may be “cost-effective” in terms of skill development, student and educator recruitment and retention, and in building a stronger, more supportive organizational culture in academic institutions. As more teachers and staff assume the role of a gatekeeper and talk with their colleagues about students they are concerned about, the more likely it is that they will build a culture that supports student mental health, which hopefully improves student outcomes and ameliorates the potential for teacher burnout. According to a 2018 report by the National Association of Elementary School Principals, an increase in the number of students experiencing trauma correlated directly with an increase in the number of teachers and administrators at risk for developing compassion fatigue (Elliot et al., 2018). Due to heavy workloads and hectic schedules, it is not uncommon that teachers are subject to developing mental health concerns, which contribute to teacher attrition. Therefore, administrative interventions for teachers can help them gain skills to identify, triage, and refer students in need of support.

## Conclusion

The results of this study provide hope that the large percentage of school-aged children who are experiencing psychological disorders, many who have not been identified or treated, could benefit immensely from high school educators and staff being trained as gatekeepers. The timing is critical, for students who access needed care in adolescence have better educational and mental health outcomes (Neufeld et al., 2017). The results also addressed some of the barriers to accessing mental health treatment including a lack of teacher and staff awareness and understanding of how psychological disorders might present which can influence students’ help-seeking behaviors (Loewen, 1993) as well as flexibility in how the intervention is delivered, as it is well suited to remote platforms. Finally, the enormous global impact of COVID-19 on student mental health, especially those with pre-existing conditions such as anxiety and mood disorders, is overwhelming. Schools are well placed to identify and treat students suffering from the psychological consequences of COVID-19.

There are several advantages of learning through online virtual role-play simulations that include (1) the lower cost and improved logistics when compared to organizing live skill practice and assessment sessions with trained actors (especially as this is taught on a virtual platform), (2) addressing the challenge of standardizing the learning experience, (3) reducing the discomfort participants often experience in live role-playing in a workshop setting, and (4) 24/7 availability and easy scalability. In a broader sense, *At-Risk for High School Educators* represents the impact that advances in simulation and gaming technology can have on our capability to help address serious public health concerns in a scalable manner to better support student mental health and improve access to services. Whether online or mobile, contextually rich online and risk-free learning environments can enable participants to bring their knowledge and skills into the real world to support a culture of mental health and supportive learning communities. Education and mental health research demonstrate the value of such engagement-based pedagogy (Lane & Rollnick, 2007; Sandler et al., 2014) and how online simulations, such as *At-Risk for High School Educators*, could engage large numbers of teachers in better supporting the mental health needs of their students and helping them access mental health services in a timely manner.

## Appendix 1. Gatekeeper behavior scale

**Table:**

**Preparedness**	**How would you rate your preparedness to:**	
	1. Recognize when a student’s behavior is a sign of psychological distress	Very low
	2. Recognize when a student’s physical appearance is a sign of psychological distress	Low
	3. Discuss with a student your concern about the signs of psychological distress they are exhibiting	Medium
	4. Motivate students exhibiting signs of psychological stress to seek help	High
	5. Recommend mental health support services (such as the counseling center) to a student exhibiting signs of psychological distress	Very high
**Likelihood**	6. How likely are you to discuss your concerns with a student exhibiting signs of psychological distress?	Very unlikely
		Unlikely
	7. How likely are you to recommend mental health/support services (such as the counseling center) to a student exhibiting signs of psychological distress?	Likely
		Very likely
**Self-efficacy**	**Please rate how much you agree/disagree with the following statements:**	
	8. I feel confident in my ability to discuss my concern with a student exhibiting signs of psychological distress	Strongly disagree
	9. I feel confident in my ability to recommend mental health support services to a student exhibiting signs of psychological distress	Disagree
	10. I feel confident that I know where to refer a student for mental health support	Agree
	11. I feel confident in my ability to help a suicidal student seek help	Strongly agree
